# Increasing Physical Activity among Breast Cancer Survivors by Modulating Temporal Orientation with rTMS: Feasibility and Potential Efficacy

**DOI:** 10.3390/ijerph181910052

**Published:** 2021-09-24

**Authors:** Ellen Carl, Alina Shevorykin, Amylynn Liskiewicz, Ronald Alberico, Ahmed Belal, Martin Mahoney, Elizabeth Bouchard, Andrew Ray, Christine E. Sheffer

**Affiliations:** Roswell Park Comprehensive Cancer Center, Buffalo, NY 14203, USA; alina.shevorykin@roswellpark.org (A.S.); amylynn.liskiewciz@roswellpark.org (A.L.); ronald.alberico@roswellpark.org (R.A.); ahmed.belal@roswellpark.org (A.B.); martin.mahoney@roswellpark.org (M.M.); elizabeth.bouchard@roswellpark.org (E.B.); andrew.ray@roswellpark.org (A.R.); christine.sheffer@roswellpark.org (C.E.S.)

**Keywords:** breast cancer, physical activity, transcranial magnetic stimulation, delay discounting

## Abstract

Maintaining adequate amounts of physical activity is a critical component of survivorship care for women with breast cancer. Increased physical activity is associated with increases in well-being, quality of life, and longevity, but women with cancer face unique, cancer-related factors that might affect physical activity. Consistent with the Competing Neurobehavioral Decision Systems model of decision making, we proposed to decrease delay discounting and increase physical activity by stimulating the executive function system via high-frequency repetitive transcranial magnetic stimulation (HF rTMS) of the left dorsolateral prefrontal cortex (LDLPFC). This randomized, sham-controlled, double-blinded trial examined the feasibility and potential efficacy of this approach to increase physical activity in breast cancer survivors. We hypothesized that active rTMS would significantly increase the mean number of steps per day and decrease delay discounting. Participants (*n* = 30) were primarily middle-aged (M = 53.7, SD = 7.9) and white with a mean BMI and body mass indices below 40. Indicators of feasibility and limited efficacy testing were positive. Although repeated-measures ANOVA revealed no significant changes in delay discounting, generalized estimating equations (GEE) found that participants in the active condition increased their mean daily steps by 400 steps per day, while those in the sham condition decreased this by nearly 600 steps per day. These findings indicate that the continued investigation of HF rTMS for increasing physical activity among women with breast cancer is justified.

## 1. Introduction

Breast cancer is the second most common cancer among women in the United States, and as of 2021, the most common cancer globally accounting for 11.7% of all new cancer diagnoses worldwide [1]. In the United States, over 280,000 women will be diagnosed and over 43,000 women will die from breast cancer in 2021 [2]. Physical activity is one of the few modifiable factors that is consistently linked to breast cancer survival rates [3,4,5]. Post-diagnosis, higher physical activity levels are linked to a 24% reduction in breast cancer deaths and a 41% reduction in all-cause mortality as well as increases in well-being, improved quality of life, and fewer treatment-related side effects [3,6]. An increase of just 600–1000 steps per day can result in clinical improvements for individuals with other health conditions [7,8]. Maintaining adequate amounts of physical activity is a critical component of survivorship care plans for women with breast cancer.

Self-regulation is a process by which individuals inhibit immediate desires and engage in health behaviors that align with temporally extended health goals [9,10,11]. The Competing Neurobehavioral Decision Systems (CNDS) model is a conceptualization of the biobehavioral processes involved in self-regulatory decision making [12,13]. Supported by numerous behavioral and neuroimaging studies [14,15,16], the CNDS model posits that decisions are driven by the balance of activity in two neural networks: (1) the executive function network, embodied in areas of the prefrontal cortex that govern prospection and the optimization of resources, and (2) the impulsive network, embodied in areas of the limbic and paralimbic regions that mediate behavior motivated by pleasure [17,18,19,20,21]. Consistent with the CNDS model, greater activity in the executive function network is associated with greater prospection [14]. Most people prefer reinforcement received sooner rather than later because rewards are evaluated, in part, as a function of temporal proximity; however, most people, albeit to varying extents, will forgo immediate rewards in lieu of larger rewards to be gained later. Delay discounting is the degree to which one discounts the value of a reward as a function of time to its receipt [22,23] and is considered a marker for the balance of activity in the two neural networks that comprise the CNDS model [24].

As with many health behaviors, rewards gained from increased physical activity through walking are often temporally distant and realized only after expending a considerable amount of effort. This pattern of foregoing exercise because benefits are not immediately realized is consistent with patterns of impulsivity over executive function in the CNDS model. Unsurprisingly, lower DD rates are associated with higher levels of physical activity [25,26,27], but this relation was not found in a large cross-sectional study of patients with cancer [28]. Cancer survivors are faced with unique physical, financial, and psychosocial consequences from cancer diagnoses and treatments that might affect the implementation of self-regulatory processes needed to increase physical activity [29,30]. Some survivors experience a sense of a foreshortened future, thoughts of early mortality, and ongoing symptoms of post-traumatic stress [31]. A sense of a foreshortened future shifts individuals’ temporal orientation to the present, which can potentially reduce the relative value placed on planning for the future [31] and decrease the value individuals place on the temporally distant reward of long-term health.

Physical activity and self-regulation also appear to have a complex bi-directional relation [32]. For instance, lower delay discounting rates are associated with a greater likelihood of engaging in physical activity [27], but increasing physical activity can decrease delay discounting as well as improve other cognitive functions associated with self-regulation [26,33,34]. This evidence suggests that the examination of delay discounting as a target to improve physical activity among women with breast cancer might encounter nuanced challenges particular to the population and the relation between physical activity and self-regulation.

High-frequency repetitive transcranial magnetic stimulation (HF rTMS), a non-invasive brain stimulation technique, can modulate activity in specific areas of the brain resulting in behavior change [35,36]. HF rTMS of the left dorsolateral prefrontal cortex (LDLPFC), a significant component of the executive function network, has been shown to decrease delay discounting rates [37,38]; calorie consumption, eating behaviors, and body weight [39,40]; and cigarette consumption [38,41,42]. rTMS is now cleared by the Federal Drug Administration (FDA) for short-term smoking cessation (FDA K200957) and major depressive disorder. In a preliminary open-label study, HF rTMS of the LDLPFC was incidentally found to improve physical activity among individuals with depression [43].

HF rTMS of the PFC has also been shown to improve learning, memory, attention, and working memory, as well as increase regional cerebral blood flow in local and distant brain regions [44,45,46]; improve connectivity in the executive function network; and enhance performance on working memory tasks [47,48]. Proposed mechanisms of change include increasing long-term potentiation (LTP) and enhancing the fronto-striatal pathway connectivity and dopamine function [49,50]. LTP, one of the major cellular mechanisms that underlie learning and memory, is one of several mechanisms involved with increasing synaptic plasticity by enhancing neuronal signal transmission [51,52,53,54]. Finally, a systematic review of rTMS depression treatment studies suggests that rTMS of the PFC might be a promising technique for cognitive enhancement [55]. This evidence suggests that reading self-help information about increasing physical activity during rTMS might improve learning associated with the material. Consistent with the CNDS model, taking in self-help material during stimulation might enhance the applicability and retention of the material.

This randomized sham-controlled, double-blind trial examined the feasibility and limited efficacy of using a minimal dose of HF rTMS to decrease delay discounting and increase steps per day among women with breast cancer. Compared to other forms of physical activity, walking is widely accessible, requires no equipment, costs nothing, and is easily tracked. Further, walking is a low-impact form of activity that is appropriate for individuals across a variety of ages and health conditions. No physical therapy, exercise training, or treatment was provided to participants. Individuals were asked to aim for 10,000 steps per day or 150 active minutes per week. Steps and active minutes were assessed with a popular, commercially available physical activity tracker, the Fitbit Alta, worn on the wrist. Feasibility was evaluated with multiple measures of interest and engagement, acceptability, and side effects, as well as the efficacy of the blind trial [56]. We hypothesized that active rTMS would increase the mean number of steps per day compared with sham rTMS. We also hypothesized that active rTMS would reduce DD rates more than the sham rTMS.

## 2. Materials and Methods

### 2.1. Participants

Recruited via community events, flyers, and social media, eligible participants were English-speaking, right-handed adult women with body mass index (BMI) < 40 and who had been diagnosed with breast cancer and completed primary treatment. Hormone therapies aimed at preventing recurrence were not exclusionary. All participants were required to have access to a smartphone capable of syncing with the Fitbit smartphone application, were motivated to increase physical activity, provided negative urine screens for drugs of abuse and pregnancy, and passed the Transcranial Magnetic Stimulation Adult Safety and Screening Questionnaire (TASS) [57]. Exclusion criteria were: personal medical history that would increase risk for rTMS (e.g., epilepsy, aneurysm, and medications that lower seizure threshold); contraindications for a magnetic resonance image (MRI) of the head (e.g., metal implants, pacemakers, and claustrophobia); uncontrolled psychiatric disorders (e.g., major depressive disorder, bipolar disorder, or schizophrenia spectrum disorder); abnormal findings on the MRI that affect participant safety (e.g., brain metastasis); ongoing treatment (besides hormone therapy) for breast cancer; and planning to become pregnant in the next 6 months. Of the 159 potential participants who were screened, 35 met all enrollment criteria, and 30 were enrolled. The five who were eligible but not enrolled, 2 did not attend their MRI appointment, and 3 did not attend the first treatment session.

### 2.2. Equipment and Materials

rTMS was delivered with the Magstim Super RAPID^2^ PLUS1 system with Magstim 70 mm Double Air Film Active and Sham Figure of 8 Coils. The Brainsight neuro-navigational system (Rogue Research, Inc., Montreal, QC, Canada) was used for precise placement of rTMS active and sham coils. The sham technique included focal electrical stimulation delivered by the DS3 Isolated Stimulator (Digitimer Ltd., Welwyn Garden City, UK). Participants received Fitbit Alta HR (Fitbit, San Francisco, CA, USA) wearable activity trackers.

### 2.3. Procedure

Participants were screened via telephone and then in-person. In-person screening included urine drug and pregnancy tests. All participants provided informed consent and a baseline assessment. To move forward, all participants were required to secure medical clearance from their physicians and undergo a structural MRI of the brain (1.5 T or 3 T, no contrast, 1 × 1 × 1 mm). Immediately prior to the first rTMS session, participants were instructed on how to use the Fitbit Alta HR and the Fitbit application and were randomized 1:1 into the active or sham condition. Participants were advised to strive for 10,000 steps per day and 150 active minutes per week and provided with psychoeducational materials about increasing physical activity. The Motor Threshold (MT) was assessed immediately prior to the first and fifth rTMS sessions. The MT is a well-established method for standardizing the intensity of stimulation. MT is defined as the minimum stimulation intensity needed to evoke a motor evoked potential (MEP) of 50 μV from the abductor pollicis brevis muscle in 3 out of 6 trials.

Participants received 8 active or sham rTMS sessions as per random assignment, one session per day, 4 days per week, for 2 weeks. Each participant was randomized 1:1 to the active or sham rTMS arms in permuted blocks of 5 participants each. The randomization was not stratified. Each session delivered 900 pulses of 20 Hz rTMS at 110% of the MT (45 20-pulse trains of 1s duration with an inter-train interval of 20 s). Total stimulation time was approximately 16 min. Participants read the psychoeducational materials during the rTMS sessions and were instructed to continue reading the material at home between sessions. Twelve weeks after the first rTMS session, participants returned to the laboratory for an outcome assessment. Participants were compensated USD 25 for the baseline and MRI sessions, USD15 for each rTMS visit, and USD 25 for the final outcome assessment (OA). Participants were invited to keep their Fitbits. Parking was validated for each visit.

The target stimulation site was the left dlPFC, situated in the middle of the frontal gyrus in the lateral part of Brodmann Area (BA) 9, near BA 46. A fiducial marker (vitamin E capsule) was established during the MRI of the head at the AF3 electrode position in the International 10-10 system for EEG electrode placement [58]. This marker was used to guide coil placement in the Brainsight (Rogue Research, Inc., Montreal, QC, Canada) neuro-navigation system. Specifically, the coil was positioned over the fiducial marker, handle pointing backward at a 45° angle with respect to the parasagittal line, and monitored throughout the session [59].

Staff involved in rTMS sessions were trained for 6 months prior to engaging with participants. The participant and the technician who delivered the rTMS were blind to conditions. All participants were prepared in the same manner, regardless of condition. After preparing for stimulation, the participant and technician left the room together. During their absence, a senior research associate attached the active or sham coil and enabled or disabled the DS3 Isolated Stimulator (Digitimer Ltd., Welwyn Garden City, UK) per the random assignment. Following a uniform 4 min, the technician and participant re-entered the room and stimulation was initiated. As part of the sham technique, electrical pulses, triggered by the TMS controller, coincided precisely with the clicking sounds of the sham coil.

### 2.4. Measures

Demographic and self-reported reports of physical activity were collected during the baseline assessment. Clinical, self-regulation, and body composition measures, described below, were collected during the baseline and 12-week outcome assessments. Positive and negative affect levels were measured using the 20-item Positive and Negative Affect Schedule (PANAS; α = 0.80) [60]. The 40-item State-Trait Anxiety Inventory (STAI; α = 0.85) was used to assess anxiety, tension, apprehension, nervousness, worry, and heightened activation of the autonomic nervous system [61]. Depressive symptoms in the past week were assessed with the Center for Epidemiological Studies Depression Scale (CES-D; α = 0.87) [62]. The 4-item Perceived Stress Scale (PSS-4; α = 0.82) was used to assess stress level over the past month [63]. Motivation and self-efficacy for increasing activity were assessed on a 0–10 scale where 0 is “not at all” and 10 is “the most ever” in response to: “How much do you want to increase exercise?” and “How confident are you that you can increase exercise?”

Delay discounting was assessed using the 5-Trial Adjusting Delay Discounting task [64]. Respondents were asked on the first trial whether they would prefer half the amount now (e.g., USD 50) or the full amount (e.g., USD 100) in three weeks. If the immediate option was selected, then the second trial shortened the delay to one day (e.g., USD 50 now or USD 100 in one day). If the delayed option was selected in the first trial, then the second trial lengthened the delay (e.g., USD 50 now or USD 100 in two years). Delays on all subsequent trials were adjusted based on responses from the preceding trial. The output was expressed as the natural logarithm of k in Mazur’s hyperbolic discounting model, with k increasing as the preference for smaller sooner rewards increases [65]. The 24-item Behavioral Inhibition System/Behavioral Activation System (BIS/BAS; α = 0.81) questionnaire was used to assess behavioral inhibition (BIS), which corresponds to motivation to avoid aversive outcomes, and behavioral activation (BAS), which corresponds to motivation to approach goal-oriented outcomes [66]. The 30-item Barratt Impulsiveness Scale (BIS; α = 0.85) was used to assess impulsiveness [67]. The BIS total score consists of three impulsiveness subscales: motor, cognitive or attentional, and non-planning. Individual differences in self-control were measured with the 13-item Brief Self-Control Scale (BSCS; α = 0.81) [68]. The 31-item Short Self-Regulation Questionnaire (SSRQ; α = 0.95) was used to measure the ability to regulate behavior to achieve one’s goals. Behavioral activation and cognitive restructuring skills were measured with the 16-item Cognitive-Behavioral Therapy Skills Questionnaire (CBTSQ; α = 0.88) [69]. Higher scores on each of these measures correspond to greater levels of self-regulation.

Measures of body composition included height, measured on a stadiometer; weight and body composition, measured via a Tanita scale; waist and hip circumferences, measured with Gulick anthropometric tape; and blood pressure, measured with an Omron HEM-907XL blood pressure monitor (Omron, Kyoto, Japan). Physical activity was tracked continuously for 12 weeks from the first rTMS session. Activity measures included the number of steps and minutes of physical activity per day.

#### 2.4.1. Feasibility

The feasibility of using rTMS to increase physical activity among women with breast cancer was assessed in multiple ways. A CONSORT diagram was used to examine the feasibility of recruitment and retention. Perceived willingness to engage in and complete all the procedures, intervention acceptability, side effects, and the effectiveness of the blind trial were assessed at each rTMS session. Actual willingness and engagement were assessed with the number of rTMS sessions attended and the number of days participants wore their Fitbits. The minimum criterium for feasibility of efficacy testing was ≥45% session attendance [56]. Perceived willingness and engagement were assessed by asking “On a scale of 0–10, where 0 = not at all and 10 = most possible, how willing are you to engage in, and complete, all the procedures in this study?” Acceptability was assessed by a question after each rTMS session “On a scale of 0–10, where 0 = not at all and 10 = most possible, how acceptable was this intervention?” and collecting reasons for missed sessions, refusal, and withdrawal. Side effects were assessed after each rTMS session. Perceived research burden was assessed with the Perceived Research Burden Assessment (PeRBA) [70]. Participant blinding was assessed by having participants guess the condition (active/sham/no idea) immediately after every stimulation session [71].

#### 2.4.2. Limited Efficacy Testing

The primary outcome measure was the change in the number of daily steps from the mean number of daily steps recorded during week one of stimulation. The secondary outcome measure was change in delay discounting of USD 100 and USD 1000 from baseline to the 12-week outcome assessment.

### 2.5. Data Analysis Plan

All analyses were conducted in IBM SPSS, Version 23 [72]. Descriptive analyses, independent measures t-tests, and Chi-square tests provided details about baseline characteristics, session attendance, side effects, reasons for refusal, and the adequacy of the blind trial. Analysis of variance (ANOVA) and Chi-square analyses were used to examine baseline differences in participant characteristics between conditions. Success of the blind trial was assessed by calculating the total number of correct guesses divided by the total number of TMS sessions across all participants. Delay discounting rates were standardized by calculating the natural log of k (lnk). Body Mass Index (BMI) was calculated using the standard formula:

(1) $$\frac{weight\;\left(lb\right)}{height\;{\left(in\right)}^{2}}\;\times \;703$$

The mean number of daily steps during the first week of the study established the baseline number of steps per day. Independent-samples t-tests were used to examine differences in the number of days participants wore their Fitbits (Fitbit, San Francisco, CA, USA). Results were reported in terms of means with standard deviations, F-tests, and *p*-values (alpha = 0.05).

Generalized Estimating Equations (GEE) with an unstructured (general) correlation matrix were used to analyze changes in steps per day [73,74]. GEE was selected because it controls for highly correlated repeated-outcome measures, such as steps per day. Condition (active or sham) was entered as the independent variable and age and BMI were entered as covariates to account for differences in physical activity and delay discounting associated with these factors [27,75]. Time was included as a repeated-measures factor. Repeated-measures ANOVA was used to examine changes in delay discounting rates, perceived willingness to participate, acceptability of the intervention, and other self-regulation measures from baseline to the 12-week outcome assessment. Condition was entered as the between-subjects factor and time (baseline, 12-week outcome assessment) as the within-subjects factor. To prevent violations of the sphericity assumption with repeated-measures data, all main effects and interactions were reported as significant after the Greenhouse–Geisser correction. A paired samples t-test was used to examine changes in post- versus pre-delay discounting rates.

## 3. Results

### 3.1. Participants

Participants were primarily middle-aged and white with some college education. The mean BMI and percent of body fat were 31.0 (SD 5.1) and 41.7 (6.7), respectively, which are within the range categorized as obese. Participants self-reported 65.6 (SD 91.9) minutes of moderate to vigorous physical activity per week during the baseline assessment; see Table 1. Significant differences between conditions were found on employment status and the SSRQ. Participants in the sham condition were more likely to be retired (*n* = 4) or unemployed (*n* = 1), while participants in the active condition were more likely to be employed (*n* = 15). Participants in the active condition scored slightly lower on the SSRQ compared to participants in the sham condition (M = 92.9 (SD 4.6) vs. M = 98.7 (SD = 9.2) *t*(28) = 2.09, *p* < 0.05, but the differences were not clinically significant. During the first week of stimulation (baseline activity), participants averaged 6073 (SD 2447.4) steps per day and 127.4 (SD 105.9) minutes of moderate to vigorous physical activity.

### 3.2. Feasibility

**Figure 1:**
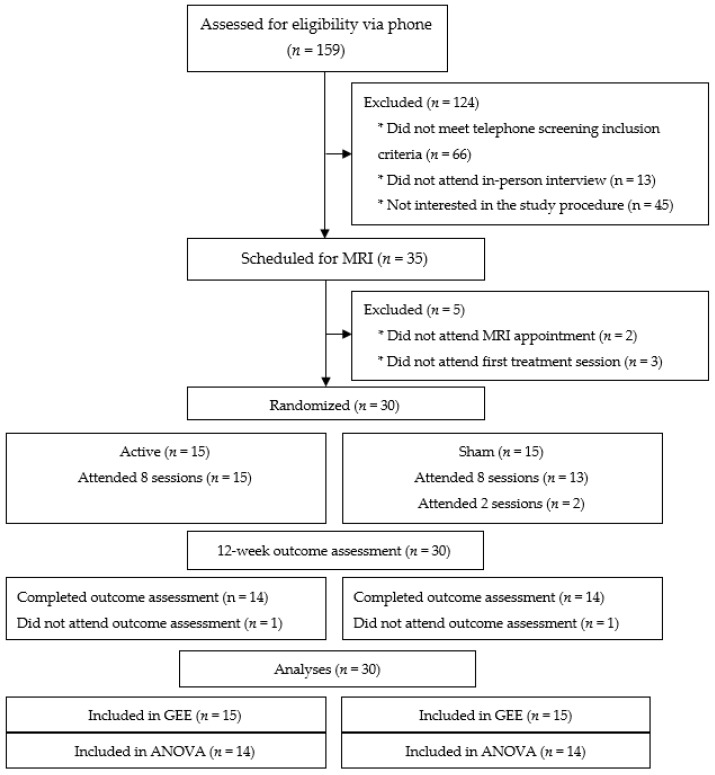
CONSORT diagram.

See CONSORT diagram (Figure 1) for recruitment and retention details.

Across conditions, 95.0% (19.0%) of sessions were completed, well above the 45% threshold needed for feasibility. Nearly all (93.3%; 28/30) participants attended all eight stimulation sessions. Condition was unrelated to perceived willingness to participate (*F*(7,182) = 0.35, *p* = 0.84) or acceptability of the intervention (*F*(7,182) = 0.64, *p* = 0.56) across the TMS sessions. There were no adverse events experienced during rTMS. Side effects were infrequent, mild, and resolved prior to the participant leaving the lab. One person experienced a mild headache, and one person experienced some mild scalp pain after one rTMS session. One individual reported a decrease in alcohol consumption. Some participants reported a change in medications related to treatments primarily or secondarily related to cancer survivorship. The success of the blind trial was established by calculating the total number of times participants correctly guessed their condition following TMS (*n* = 41) divided by the total number of TMS sessions (*n* = 228). Participants guessed the condition correctly 17.9% of the time, indicative of a successful blinding procedure for both conditions.

No differences were found in the number of sessions attended in the active (M = 8, SD = 0) and sham (M = 7.2, SD = 2.1) conditions, *t* = −1.5, *p* = 0.20. No participants withdrew, although two were lost to follow up (1 each in the active and sham conditions). Reasons for missed appointments (TMS and outcome sessions) included change to an exclusionary medication (Bupropion, *n* = 1, sham) and an incompatible schedule (*n* = 1, active; *n* = 2, sham). No differences were found between the active and sham conditions in the number of days participants wore their Fitbits (M = 72.9, SD = 22.5 vs. M = 64.5, SD = 25.2; *t*(28) = −0.97, *p* = 0.34). Perception of total research burden, as measured by the PeRBA, did not differ significantly between active and sham coils over time *F*(1,26) = 0.70, *p* = 0.41. There were no perceived differences in logistical (*F*(1,26) = 0.33, *p* = 0.57), psychological (*F*(1,26) = 1.1, *p* = 0.31), or physical (*F*(1,26) = 0.41, *p* = 0.53) burden between active and sham coils over time.

### 3.3. Limited Efficacy Testing

**Figure 2:**
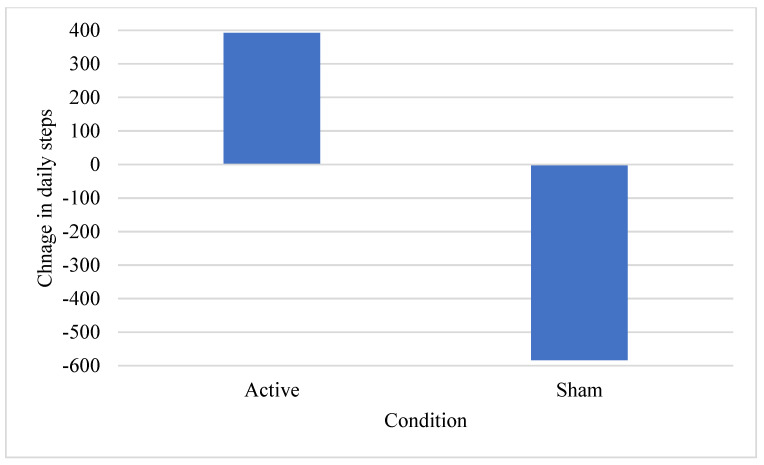
Change in mean daily steps per day by condition.

**Figure 3:**
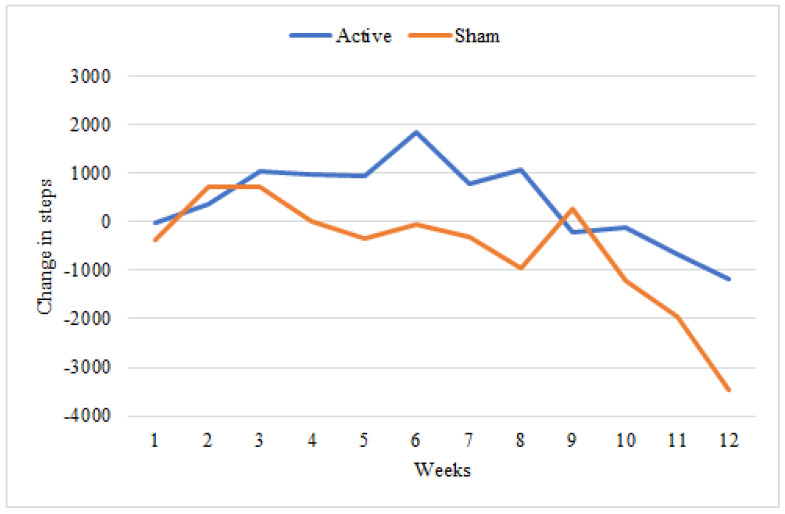
Weekly mean change in number of daily steps.

GEE revealed a significant main effect of condition on the change in mean steps per day, χ^2^ = 4.6, *p* = 0.03; a significant main effect of time χ^2^ = 1120.8, *p* < 0.001; and a significant interaction between time and condition, χ^2^ = 105.4, *p* < 0.001. Participants in the active condition increased their mean daily steps by 400 steps per day over 12 weeks of participation, while those in the sham condition decreased by nearly 600 steps per day; see Figure 1. Age was a significant covariate (χ^2^ = 71.9, *p* < 0.001), but BMI index was not (χ^2^ = 1.2, *p* = 0.28). Greater age was associated with smaller changes in steps per day B = −112.5, SE = 13.3. Participants who received active rTMS increased the number of steps per day more than participants who received sham rTMS (B_active_ = 350.1, SE = 490.7). The increase in steps also appeared to be maintained after active rTMS for weeks 3–8. Although the increase was not sustained longer term (weeks 9–12), the reduction in steps per day compared to baseline appeared to be smaller for active vs. sham rTMS; see Figure 2 and Figure 3.

### 3.4. Delay Discounting

**Figure 4:**
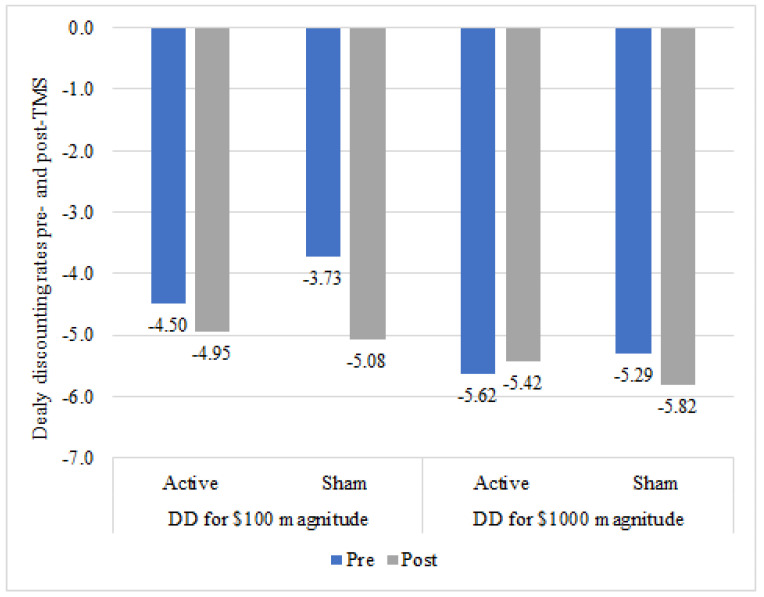
Pre- and post-TMS intervention delay discounting values by condition.

Repeated-measures ANOVA revealed no significant main effect of condition on delay discounting rates (USD 100: F = 0.58, *p* = 0.48; USD 1000: F = 0.01, *p* = 0.94). A main effect of time was found for USD 100 magnitude, F = 10.34, *p* < 0.01, η^2^ = 0.30, but not for USD 1000 magnitude, F = 0.77, *p* = 0.39. No interactions between time and condition (USD 100: F = 2.59, *p* = 0.12; USD 1000: F = 3.97, *p* = 0.06) were found, although the interaction between time and condition for USD 1000 approached significance; see Figure 4.

Paired-samples t-tests revealed that, across conditions, delay discounting rates significantly decreased for the USD 100, but not for USD 1000 magnitudes (USD 100: *t* = −3.12 *p* < 0.01, M_pre_ = −4.11 vs. M_post_ = −5.01; USD 1000: *t* = −0.83, *p* = 0.41, M_pre_ = −5.46 vs. M_post_ = −5.62).

### 3.5. Other Self-Regulation Measures

Repeated-measures ANOVAs revealed no significant condition-related changes between baseline and 12 weeks in the BIS/BAS subscales of Drive (*F*(1,26) = 0.38, *p* = 0.66), Fun Seeking (*F*(1,26) = 0.76, *p* = 0.39), Reward Response (*F*(1,26) = 0.39, *p* = 0.54) or Behavioral Inhibition (*F*(1,26) = 1.04, *p* = 0.32); the total Barratt Impulsiveness Scale (BIS) score (*F*(1,26) = 0.62, *p* = 0.44), the Attentional (*F*(1,26) = 0.19, *p* = 0.66), Motor (*F*(1,26) = 0.17, *p* = 0.68), or Non-planning (*F*(1,26) = 0.13, *p* = 0.73) impulsiveness subscales; the BSCS (*F*(1,26) = 0.42, *p* = 0.52) or SSRQ (*F*(1,25) = 2.3, *p* = 0.14). No significant condition-related changes were found in behavioral skills (*F*(1,26) = 0.21, *p* = 0.65), cognitive skills (*F*(1,26) = 0.74, *p* = 0.39), or total (*F*(1,26) = 0.18, *p* = 0.68) acquisition scores on the CBTSQ.

## 4. Discussion

These findings suggest that the investigation of HF rTMS to increase physical activity among women with breast cancer is feasible and has potential efficacy. Women with breast cancer appear to be interested, engaged, and adherent to study procedures in both the active and sham conditions. Given that the sham condition provides no stimulation, we anticipated a potential for lower adherence, but that was not realized. The perceived acceptability of the intervention stayed consistently high in both conditions over time. Side effects were mild and resolved within approximately 20 min. Perceived research burden was relatively low and did not increase over time. As in previous rTMS studies, study blinding was effective [38]. Active rTMS showed greater increases in steps per day over time than sham rTMS, providing evidence for potential efficacy.

The Fitbit activity tracker served as both a measure of activity and a minimal intervention (i.e., self-monitoring) in this study, and this aspect of the study design precludes disentangling the impact of the Fitbit and the rTMS. The novelty effects for wearable fitness devices appears to be approximately 12 weeks [76], the length of this study. All participants were given the device during the same visit, during which they initiated rTMS, to control for potential variability in the use of the Fitbit prior to initiating rTMS. Baseline physical activity was subsequently established as the first week of rTMS sessions. We viewed this as a conservative approach to establishing an accurate baseline, even though the active rTMS might have potentially increased steps during this week, making it more difficult to detect condition-related changes in activity.

Contrary to our hypothesis, and inconsistent with the previous literature [37,38,77,78], we found no significant difference in delay discounting rates between conditions. We speculate that this may be due to the following: (1) The general increases in physical activity, although brief for the sham condition, could have been associated with decreased delay discounting across both conditions as a product of the design. Increased physical activity has been associated with decreased delay discounting rates [26]. Participants in both conditions were given the same physical activity goals, used the Fitbit for monitoring their activity, and received psychoeducational materials, all of which have been shown to support some increased physical activity, albeit temporarily. This speculation is partially supported by the general decrease in delay discounting across conditions found in the results. (2) The brief five-trial adjusting delay discounting task might not have been sensitive enough to detect small changes in delay discounting rates in a small sample of participants. (3) Changes in delay discounting could have occurred during the study, but were not assessed between baseline and the 12-week outcome assessment. (4) Cancer survivors might hold a unique valuation of future rewards [28]. Delay discounting rates for cancer survivors might be influenced by the nature of the serious diagnosis and more difficult to change or more dissociated from physical activity levels than among individuals without cancer. This speculation is supported by findings from a large cross-sectional study of delay discounting and health behaviors among cancer survivors [28]. (5) The sample size was too small to provide enough power to detect a difference in delay discounting rates. (6) Finally, eight sessions of HF rTMS might not be a large enough dose to produce a significant change in delay discounting rates for these participants.

These findings suggest that the examination of rTMS as an intervention to increase physical activity among women with breast cancer is promising. Given the positive effects of increased physical activity on cancer mortality [3], as well as the recent finding that rTMS paired with physical activity can improve exercise-related neuroplasticity [79], further investigation of rTMS as an intervention to improve physical activity in this vulnerable group is justified. Future research should utilize multiple delay discounting measures, include more frequent outcome assessments, include a variety of rTMS dosing strategies, take advantage of the neuroplasticity provided by HFrTMS by providing more intensive behavioral intervention in conjunction with stimulation, examine the approach among other cancer survivors and the general population, and determine the dose–response relationship between stimulation parameters and physical activity outcomes.

This novel approach to increasing physical activity among women with breast cancer had a few limitations and several methodological strengths. Limitations include a small sample size, which possibly interfered with our ability to detect differences between groups; the lack of a natural baseline (pre-rTMS) measure of activity; a convenience-based sample with limited diversity which may not be reflective of the broader cancer patient population; and the use of a single outcome assessment, which limited our ability to examine delay discounting and other measures throughout the study period. Strengths include continuous monitoring of physical activity, which removes recall bias associated with self-reported measures, and the use of multiple measures specifically relevant for determining the feasibility of continued examination of this approach.

## 5. Conclusions

rTMS is feasible, well tolerated, and shows potential efficacy as an intervention to improve physical activity among breast cancer survivors. Future studies should further develop this relationship and compare the rTMS intervention to other therapeutic rehabilitation approaches. These results, in conjunction with previous research observing the positive effects of increased physical activity on cancer mortality and enhancements in the long-term effects of physical exercise associated with rTMS, suggest that further investigation of the benefits of rTMS as a tool to enhance physical activity is needed.

## Figures and Tables

**Table 1:** Participant characteristics at baseline.

Variable	Range or Categories	Percent (*n*) or Mean (SD)		
		Total (30)	Active (15)	Sham (15)
Age		53.7 (7.9)	55.2 (8.2)	52.1 (7.7)
Race	Caucasian or White	93.3 (28)	93.3 (14)	93.3 (14)
	African American or Black	6.7 (2)	6.7 (1)	6.7 (1)
Partnered status	Partnered	66.7 (20)	66.7 (10)	66.7 (10)
Annual household income	≤USD 34,999	13.3 (4)	13.3 (2)	13.3 (2)
	USD 35,000–USD 74,999	23.3 (7)	20.0 (3)	26.7 (4)
	≥USD 75,000	63.3 (19)	66.7 (10)	60.0 (9)
Education	High school	13.3 (4)	13.3 (2)	13.3 (2)
	College	66.7 (20)	66.7 (10)	66.7 (10)
	Graduate school	20.0 (6)	20.0 (3)	20.0 (3)
Employment status *	Full Time	76.3 (22)	53.3 (8)	93.3 (14)
	Part Time	10.0 (3)	13.3 (2)	6.7 (1)
	Retired	13.3 (4)	26.7 (4)	-
	Unemployed	3.3 (1)	6.7 (1)	-
Health insurance	Medicaid	3.3 (1)	6.7 (1)	-
	None	6.7 (2)	6.7 (1)	6.7 (1)
	Private	90.0 (27)	86.7 (13)	93.3 (14)
Self-reported minutes of moderate to vigorous physical activity per week	0–300	65.6 (91.9)	101.0 (105.9)	30.1 (59.9)
PANAS	Positive	34.1 (7.4)	34.3 (7.2)	34.0 (7.8)
	Negative	13.1 (4.3)	12.6 (4.9)	13.7 (3.6)
STAI	State	28.2 (7.7)	26.5 (6.1)	29.9 (8.9)
	Trait	33.3 (8.6)	32.2 (8.9)	34.5 (8.4)
CES-D	0–60	10.2 (8.5)	9.3 (8.6)	11.1 (8.6)
PSS-4	0–16	4.8 (2.6)	4.6 (2.9)	5.0 (2.3)
Motivation to increase physical activity	0–10	9.0 (1.4)	8.9 (1.5)	8.4 (1.2)
Efficacy to achieve 10,000 steps per day	0–10	8.1 (1.9)	8.1 (2.3)	8.1 (1.8)
Efficacy to achieve 150 min of moderate to vigorous activity per week	0–10	8.2 (1.8)	8.5 (1.6)	7.9 (1.9)
Delay discounting (USD 100) logk		−3.9 (1.6)	−4.4 (0.4)	−3.6 (1.6)
Delay discounting (USD 1000) logk		−5.3 (1.1)	−5.5 (1.4)	−5.2 (0.9)
BIS/BAS	BAS: Drive	10.8 (2.4)	10.9 (2.2)	10.8 (2.7)
	BAS: Fun Seeking	11.5 (1.5)	11.4 (1.3)	11.5 (1.6)
	BAS: Reward Response	17.4 (1.9)	17.1 (2.3)	17.7 (1.4)
	BIS	20.1 (3.4)	19.9 (3.8)	20.3 (3.1)
BIS	Attentional	16.1 (3.1)	16.0 (3.8)	16.3 (2.4)
	Motor	23.0 (2.4)	23.3 (2.1)	22.8 (2.7)
	Non-planning	23.5 (2.9)	22.8 (2.4)	24.1 (3.4)
	Total	62.6 (6.6)	62.1 (6.2)	63.2 (7.1)
BSCS		44.3 (7.3)	46.0 (7.4)	42.7 (7.0)
SSRQ *		95.8 (7.8)	92.9 (4.6)	98.6 (9.4)
CBTSQ	Behavioral	24.9 (3.3)	25.1 (3.4)	24.8 (3.3)
	Cognitive	27.9 (5.6)	28.7 (4.1)	27.2 (6.9)
	Total	52.9 (7.3)	64.7 (2.1)	52.0 (8.2)
Height (inches)		64.7 (2.7)	64.7 (2.1)	64.7 (3.3)
Weight (pounds)		185.1 (34.8)	185.9 (35.8)	184.2 (34.9)
Body fat percentage	0–100	41.7 (6.7)	41.6 (7.1)	41.7 (6.5)
Muscle Mass (pounds)		99.4 (11.0)	101.1 (11.9)	100.2 (10.4)
Bone Mass (pounds)		5.4 (0.6)	5.4 (0.6)	5.4 (0.6)
Visceral Fat		10.2 (3.1)	10.5 (2.9)	10.0 (3.5)
Body Mass Index		31.0 (5.1)	31.1 (5.2)	30.9 (5.1)
Waist Circumference		42.8 (16.1)	45.6 (22.3)	39.9 (4.8)
Hip Circumference		47.9 (13.9)	50.3 (19.2)	45.6 (4.0)
Systolic Blood Pressure		123.2 (17.2)	125.5 (20.3)	120.8 (13.7)
Diastolic Blood Pressure		78.8 (7.9)	78.9 (9.3)	78.7 (6.6)

* < 0.05; PANAS: Positive and Negative Affect Schedule; STAI: State-Trait Anxiety Inventory; CES-D: Center for Epidemiological Studies Depression Scale; PSS-4: Perceived Stress Scale; BIS/BAS: Behavioral Inhibition System/Behavioral Activation System; BIS: Barratt Impulsiveness Scale; BSCS: Brief Self-Control Scale; SSRQ: Short Self-Regulation Questionnaire; CBTSQ: Cognitive-Behavioral Therapy Skills Questionnaire.

## Data Availability

The data presented in this study are available on request from the corresponding author. The data are not publicly available to retain participant privacy.
